# Association Between Maternal Dietary Fatty Acid Intake and Fatty Acid Composition of Placental Phospholipids

**DOI:** 10.3390/nu17152394

**Published:** 2025-07-22

**Authors:** Liliana Ladino, Hans Demmelmair, María Teresa Segura, Mireia Escudero-Marin, Veit Grote, Berthold Koletzko, Cristina Campoy

**Affiliations:** 1 Department Pediatrics, Dr. von Hauner Children’s Hospital, Division of Metabolic and Nutritional Medicine, LMU University Hospitals, LMU—Ludwig-Maximilians-Universität München, 80337 Munich, Germany; lladino@cienutrition.org (L.L.); hans.demmelmair@med.uni-muenchen.de (H.D.); veit.grote@med.uni-muenchen.de (V.G.); 2 German Center for Child and Adolescent Health, 81377 Munich, Germany; 3 Excellence Centre for Paediatric Research (EURISTIKOS), University of Granada, 18016 Granada, Spain; maite.segura.moreno@gmail.com (M.T.S.); mireia@ugr.es (M.E.-M.); ccampoy@ugr.es (C.C.); 4 Research and Education Center on Nutrition, CIENutrition, Bogotá 110221, Colombia; 5 Department of Paediatrics, University of Granada, 18016 Granada, Spain; 6 Biosanitary Research Institute of Granada (Ibs-GRANADA), 18014 Granada, Spain; 7 CIBERESP—Epidemiology and Public Health Research Network—ISCIII, Granada’s Node, 28029 Madrid, Spain

**Keywords:** dietary intake, pregnancy, fatty acids, docosahexaenoic acid, placenta

## Abstract

**Background:** Fatty acid status during the perinatal period is important for optimal offspring growth and development. **Objectives**: We aimed to test the association between maternal fatty acid (FA) intake during the third trimester of pregnancy and the FA composition of placental phospholipids, a marker of maternal fatty acid status. **Methods**: This cohort study was performed on 54 mothers participating in the PREOBE study. Maternal dietary intake was assessed with prospective 7-day food diaries at 34 weeks of gestation. Placenta samples were collected immediately after delivery and phospholipid FA was quantified with established methods. Data were analyzed with Pearson correlations and linear regression models, with adjustment for confounding factors. **Results**: Total energy intake was 2019 ± 527 kcal/d (mean ± SD) and total fat intake of the mothers was 87 ± 35 g/day. Myristic, stearic, oleic, and α-linolenic acid intakes were modestly correlated with placental percentages, with r-values ≤ 0.33. Only docosahexaenoic (DHA) acid intake (%-energy, %-fat, and g/d) showed r-values > 0.4 for the correlation with placenta phospholipids. Intake of other fatty acids, including arachidonic acid, was not associated with the placenta percentage. Linear regression models considering confounders showed only dietary DHA intake significant associations. Total fat intake did not interfere with the association of DHA intake with placental incorporation. **Conclusions**: DHA and arachidonic acid are enriched in the placenta, but only placental DHA content seems modifiable by maternal dietary DHA intake.

## 1. Introduction

Maternal nutrition and metabolism during pregnancy can affect both fetal growth and long-term offspring health [1,2]. The supply and metabolism of the omega-3 docosahexaenoic fatty acid during pregnancy has received particular attention. The preferential materno-fetal transfer of docosahexaenoic acid (DHA) mediated by specific binding and transport proteins has been shown for docosahexaenoic acid but not for other fatty acids [3]. Reduced placental MFSD2A transporter expression is related to decreased DHA in the cord blood of women with treated gestational diabetes [3,4]. Maternal DHA status in pregnancy has been associated with fetal growth, offspring neurodevelopment [5], immune system development and allergy risk [6], and obesity [7]. Several randomized controlled intervention trials have established a cause-and-effect relationship between an increased DHA intake or status in early pregnancy and a marked reduction in the risk of preterm and particularly early preterm birth [8].

Several previous studies investigated associations between maternal DHA intake during pregnancy and its content in maternal plasma and erythrocytes [9], umbilical cord blood plasma and erythrocytes [10], and postnatal infant plasma levels [11,12]. The studies agree with positive associations between maternal dietary intake and levels of fatty acid (FA) in the maternal circulation, and most of the studies also indicate positive associations with infant plasma and red blood cell levels.

Placental tissue FA has also been studied, with an emphasis on long-chain polyunsaturated fatty acids (LC-PUFAs), focusing on maternal pre-pregnancy status, obesity, diabetes, and pregnancy complications, while there was little consideration of maternal diet. Gestational diabetes mellitus (GDM) was associated with increased LC-PUFA percentages in placental phospholipids (PL) [13]. In vitro studies confirmed the influence of maternal glycemia on the incorporation of DHA and other fatty acids into PL in placenta samples [14,15].

The incorporation of DHA into PL has received specific attention, as there are good indications that DHA content influences the placental activity of proteins related to FA transport and oxidation [16]. To a certain extent the FA composition of most body compartments is associated with dietary FA intake. However, possible dietary effects on placental FA and specifically DHA and arachidonic acid (ARA) contents have not been characterized. We studied the associations between maternal dietary FA intake during the third trimester of pregnancy and the FA levels in placental PL at parturition in a cohort of Spanish women.

## 2. Materials and Methods

### 2.1. Study Design and Subjects

The current study was performed on pregnant women participating in the PREOBE study *“Role of Nutrition and Maternal Genetics on the Programming of Fetal Adipose Tissue Development”* (www.ClinicalTrials.gov identifier: NCT01634464) coordinated by the University of Granada, Spain. Detailed information was previously published elsewhere [17]. Briefly, PREOBE is an observational cohort study aimed at assessing the long-term effects of body weight and diabetes during pregnancy on the growth and development of offspring. A total of 331 women with uncomplicated singleton pregnancies meeting the inclusion criteria were recruited from 2008 to 2012 at gestational weeks 12 to 20 and divided into four study groups based on pregestational body mass index (BMI) and GDM (healthy normal weight (18.5 ≤ BMI < 25), healthy overweight (25 ≤ BMI < 30), healthy obese (BMI ≥ 30), or GDM (BMI ≥ 18.5)). The analysis was based on placental samples together with dietary intake from 54 women.

At the screening visit, pre-pregnancy BMI, maternal age, education, socioeconomic status, and smoking were assessed. At 34 weeks of gestation a prospective food protocol was applied and physical activity was evaluated by self-reporting of the women (low active, active, very active). Gestational weight gain (GWG) was calculated using self-reported weight at delivery and medical records on weight prior to or during the first trimester. The occurrence of GDM according to the National Diabetes Data Group criteria [18], placental weight infant sex, and birth weight were obtained from medical records.

The Ethics Committees of the University of Granada and of the Clinical University Hospital San Cecilio and the Mother-Infant University Hospital of Granada, approved this study. All women provided written consent after receiving a full study explanation and before any study procedure was performed.

### 2.2. Assessment of Maternal Dietary Intake

At 34 weeks of gestation the quality of the mothers’ diet was assessed using a food frequency questionnaire (FFQ) and the quantity of the mothers’ diet was obtained using standardized 7-day dietary records [19]. A detailed explanation about how to complete the records was given to the mothers during their 24-week visit. Information about the date (weekday, weekend day, or holiday), time of the meal (breakfast, lunch, dinner, or anything else), food preparation or recipe of the dishes, and portion size (cups, tablespoon, or teaspoon) was collected. Trained research nutritionists checked both the information in the FFQ and the dietary records for completeness and accuracy. For this study, only the dietary records were analyzed. Dietary records with less than 3 complete days recorded (2 week days and 1 holiday or weekend day) were considered as non-evaluable.

The intake of energy (kcal/day), macronutrients (carbohydrates, protein and fat), and FA were calculated using the Spanish DIAL 1.0 software (https://www.alceingenieria.net/nutricion.htm) accessed during 2013. DIAL software is based on different food composition databases, with a wide compilation of standardized recipes and mixed dishes typical of Spanish foods. DIAL software enabled quantification of the dietary intake of myristic acid (C14:0), palmitic acid (C16:0), stearic acid (C18:0), palmitoleic acid (C16:1), oleic acid (C18:1*n*-9), linoleic acid (C18:2*n*-6, LA), α-linolenic acid (C18:3*n*-3, ALA), arachidonic acid (C20:4*n*-6, ARA), eicosapentaenoic acid (C20:5*n*-3, EPA), and docosahexaenoic acid (C22:6*n*-3, DHA). The adequate reference values according to the dietary reference intake [20] for the percentage of the total energy intake (%E) from carbohydrates was 45–65%E, from protein was 10–35%E, and from fat was 20–35%E.

### 2.3. Placenta PL Fatty Acids

Placenta tissue samples were collected immediately after delivery, washed, frozen at −80 °C, and analyzed as previously described [21]. Tissue was homogenized and after the addition of internal standard (C15:0) lipids were extracted using a modification of the Folch procedure. The PL fraction was isolated by thin layer chromatography. FA methyl-esters were obtained by reaction with methanolic HCl and quantified by capillary gas/liquid chromatography using a HP-5890 series II gas chromatograph (Hewlett-Packard, Waldbronn, Germany) equipped with a BPX70 column (SGE, Weiterstadt, Germany, length: 25 m, inner diameter: 0.22 mm) [21]. FA in placental PL was identified by comparison with authentic standards (Nu-Chek-Prep, Elysian, MN, USA). FA in placental PL was quantified as mg/g wet tissue and results expressed as mg of total FA/g and as weight-% of the total analyzed FA.

### 2.4. Statistical Analysis

Descriptive data were obtained from all continuous variables and expressed by mean ± standard deviation, maximum, and minimum. For non-continuous variables the values were shown as number of cases and (percentage of the total sample). Pearson’s correlation analysis between maternal dietary FA intake at 34 weeks of pregnancy, and corresponding total FA in placental PL, and individual FA% of the total FA analyzed (dependent variables), was performed. To assess the influence of potential confounding factors on the significant associations of FA in placental PL with maternal FA intake, individual linear regression models were performed, including potential confounders such as maternal age, various markers of socioeconomic status (employment, residence (urban or city), number of family members, and education level (university, technical school, or elementary school)), smoking, pre-pregnancy BMI, GWG, GDM, placenta weight, infant sex, infant birthweight, total dietary energy, and fat intake. In this exploratory study, significance was accepted at *p* < 0.05 without consideration of multiple testing. Statistical analyses were performed using the SPSS statistical software package (version 28.0; IBM SPSS Inc., Chicago, IL, USA) and the R Project for Statistical Computing (version 3.3.3).

## 3. Results

In this study, 54 women were included. Twenty-four of the women were classified as healthy and normal weight, nine as overweight, and six as obese. Fifteen of the participants developed GDM (twelve normal weight and three overweight). Characteristics of the studied mothers and babies (53% male) are shown in Table 1. In addition, all infants were born at term and with an adequate gestational age. The majority of the women had a university education (43%), followed by elementary and technical school (31% and 26%). They were mostly employed (72%) and lived in rural areas (52%). The physical activity of the women was mostly self-described as active (49%), whereas fewer women reported low active (39%) or very active (12%). Prior to pregnancy, 72% of participants reported being non-smokers, indicating it was the predominant behavior. Nevertheless, 28% reported smoking, representing a notable minority.

### 3.1. Maternal Dietary Intake

The maternal dietary intake at 34 weeks of gestation is shown in Table 2. Energy intake was 2019 ± 527 kcal/d (mean ± SD). All the women achieved the recommended percentage intake from proteins, but only 25 and 19 women achieved the recommended percentage from carbohydrates and fats, respectively. Regarding the distribution of fatty acids, saturated fatty acid (SFA) and monounsaturated fatty acid (MUFA) intake above 10%E was met in 39 and in 54 women, respectively. Most women had an LA intake below 5%E (76%) or below 13 g/day (79%) and an ALA intake below 0.6%E (69%) or below 1.4 g/day (63%), respectively. Most women (87%) also showed a dietary LA:ALA ratio above 10:1.

### 3.2. Placental Fatty Acid Association with Diet

Total placental PL bound FA concentration was 7.1 ± 1.3 mg/g, with C16:0 and C18:1*n*-9 as the most abundant SFA and MUFA, respectively. While percentages of essential FA were low, ARA and DHA were the most abundant *n*-6 and *n*-3 FA, respectively (Table 3).

Concentrations of total placental PL fatty acid were not significantly related to maternal energy intake, the intake of fat or other macronutrients, or the intake of any FA. Thus, we focused further analyses on FA percentages. Saturated placental FA myristic acid and stearic acid, but not palmitic acid, correlated with maternal dietary intake, expressed as g/day. On the other hand, %E or %Fat of oleic acid and α-linolenic acid in the diet correlated with the placental percentage (Table 4).

While intakes of LA, ARA, and EPA during the third trimester of pregnancy were not significantly correlated with their corresponding percentages in placenta PL, DHA showed the closest associations between intake (expressed either as g/day, %E, and %Fat) and placental PL content (Table 4).

EPA intake was positively correlated with placental DHA in PL (r = 0.452, *p* < 0.01), while the correlations between other precursor PUFA in the diet and the respective LC-PUFA derivatives in the placenta were not statistically significant for any of the following pairs: LA-ARA, ALA-EPA, and ALA-DHA. Associations between FA dietary intakes showed no significant correlations for ALA, while LA and ARA intake (g/day) showed a correlation of 0.553 (*p* < 0.001) and the correlation between EPA and DHA in the diet was 0.944 (*p* < 0.001).

**Figure 1:**
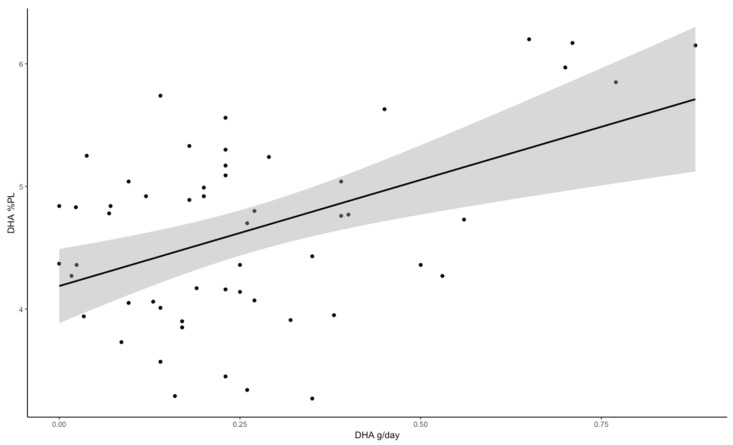
Linear positive correlation between maternal dietary docosahexaenoic acid intake (g/day) and % of docosahexaenoic acid in placental phospholipids. DHA%PL = 4.188 + 1.731 DHA g/day, r^2^adj. = 0.205; *p* < 0.01.

FA linear regression models were performed with the potentially confounding factors. Including any of the cofounders yielded non-significant models for all FAs other than DHA. The inclusion of confounders did not significantly affect the association between any of the parameters describing DHA intake with DHA percentage in placenta PL. In the various regression models, none of the considered confounders were found to be significantly associated with the DHA percentage in placental PL. In the unadjusted linear regression model, the maternal dietary DHA intake (g/day) explained 20.5% of the variance of DHA percentage in placental PL (Figure 1).

## 4. Discussion

The maternal dietary intake at 34 weeks of gestation was similar to the whole PREOBE population [17]. The total PL content of placental tissue was not related to the maternal intake of macronutrients or any specific fatty acid. For PL fatty acid composition, we found that the placental percentages of palmitic acid, palmitoleic acid, linoleic acid, arachidonic acid, and eicosapentaenoic acid were not related to the corresponding fatty acid intake, but there were modest associations for myristic acid, oleic acid, and α-linolenic acid. Highly significant associations with r-values above 0.4 independent of the way intake was calculated were only found for docosahexaenoic acid.

By applying similar quantification methods for the placentas of diabetic, obese, and healthy mothers, a total fat content of around 15 mg per g tissue has been reported [22,23]. In the PREOBE cohort we did not see differences in the placental PL or triglyceride content between normal weight, obese, and diabetic mothers and the triglyceride content was below 1 mg/g [21], which would suggest that other lipids, such as cholesterol esters and not covalently bound FA provide a major portion to total placenta FA in healthy and complicated pregnancies [21]. In the PL fraction we found no indication of lower lipid content with a higher EPA or DHA intake. Thus, the lower lipid content in the placenta with *n*-3 LC-PUFA supplementation during pregnancy observed by Calabuig-Navarro et al. probably affects other lipid classes and not PL, which are mostly membrane components [24].

For many body compartments it has been shown that the FA composition is associated with the FA intake, although differences in turnover rates and incorporation preferences limit the association [25,26]. Similarly, we found modest but significant positive associations between myristic acid, stearic acid, oleic acid, and α-linolenic acid intake and placental PL fatty acid percentages, while there were no associations for palmitic acid, linoleic acid, ARA, or EPA. A strong positive association between dietary intake and placental percentage was found only for docosahexaenoic acid.

Previous studies showed that palmitic acid dietary intake does not strongly influence its concentration in the circulation [25,26]. Likewise, we also found no such association. Thus, this study confirms the conclusion that de novo lipogenesis from carbohydrates is a major source of tissue palmitic acid [27]. Similarly, ARA levels in plasma and red blood cells have not been found related to dietary intake [25,26]. The absence of correlations between LA intake and ARA concentrations in placental PL suggests that LA dietary availability did not limit arachidonic acid endogenous synthesis in the women in the PREOBE study. Fatty acid desaturase genotypes have explained considerable portions of ARA concentration variance, without taking LA intake or blood levels into account [28,29]. Thus, synthesis capacity (not precursor availability) seems to limit ARA availability for placental incorporation. Placental *n*-6 LC-PUFA concentrations in vegetarians with minimal ARA intake are similar to those in women who are omnivorous [30], implying that ARA status is largely dependent on de novo synthesis. Analogous to our findings, the DHA content in human milk varies widely depending on fish intake, whereas milk arachidonic acid content is rather constant and not related to dietary variation [31,32,33].

Blood levels of both the essential linoleic acid and EPA are associated with dietary intake [26], and blood LA is also associated with adipose tissue content [34]. EPA shows lower levels in the placenta than in red blood cell membranes [35], which could suggest that dietary EPA availability does not limit placental tissue incorporation and thus there is no association with intake. The close correlation of EPA and DHA dietary intake is of interest, consistent with the fact that fatty fish provides both these FAs. The observed correlation between dietary EPA and placental DHA is probably due to the high correlation between EPA and DHA intake. This might even be enhanced by an EPA dependent synthesis of DHA from EPA [36] but also confirms the preferential incorporation of DHA compared with other fatty acids [37].

A supplementation study in rats showed a four times higher linoleic acid intake to induce an increased linoleic acid percentage in placental total lipids. However, the variability in the self-selected diets of the women in our study seems too small to affect the placenta’s PL linoleic acid percentage. The rather close association between dietary docosahexaenoic acid intake and placental PL content, in contrast with the findings for other fatty acids, suggests that dietary docosahexaenoic acid availability limits its incorporation into the placenta and most likely also its transfer to the fetus. Previous studies demonstrated the influence of DHA intake on DHA content pools, such as maternal and cord blood, which are regularly exchanging FA with the placenta [38,39]. Thus, our results are in line with these studies, showing an influence of diet on placental DHA concentrations. An intervention study providing 2 g of *n*-3 LC-PUFA to pregnant women from gestational week 20 onwards found increased maternal plasma EPA and DHA at delivery but no supplementation effect on these FAs in placenta or adipose tissue [40]. This adds to our observation that long-term habitual dietary intake, eventually including intermediate storage in adipose tissue, is the major determinant of tissue FA composition. A preferential placental docosahexaenoic acid incorporation and transfer has been found in vitro using the perfused placenta model and in vivo using stable isotope-labeled FA [41]. Together with our observed correlation, this suggests that the availability of DHA strongly influences its level in the feto-placental system. The docosahexaenoic acid status may be important not only for the fetal DHA level but also for further placental functions, including FA transport proteins and FA synthase and diacylglycerol O-acyltransferase 1 [23]. Of interest is the observation that intakes of DHA expressed as g/day, %E, or %fat all showed a similar high correlation with DHA in placental PL, while adjusting for energy or total fat intake did not affect the association. We found an increase in the DHA intake by 100 mg/day associated with a 0.17 percent point increase in placenta DHA percentage, independent of energy intake and total fat intake. A DHA supplementation trial during the first 12 weeks’ postpartum found that every 100 mg/day increase in maternal DHA intake induced an increment of 0.072 percent points of DHA in human milk [42]. This is the same order of magnitude that we found in the placenta in the present study and supports the validity of our findings. As milk fat contains mainly triglycerides with a low DHA percentage compared with placental tissue [43], the absolute effect size induced by a dietary change can be expected to be larger in placental tissue, which is again in line with our study observations.

Strengths and limitations. This is the first study to combine information about habitual maternal dietary FA intake in different metabolic conditions and placenta analyses in humans. Dietary intake was determined by combining the protocol data and FFQ, which strengthens the validity of the data. Specifically, PL fatty acids were analyzed, reducing the influence of placental lipid storage and acting as a better indicator of long-term diet than other lipid fractions, showing a faster incorporation of plasma FA [37]. Our study findings were strengthened by the consideration of potentially confounding factors. Our study was limited to placenta analyses. Interpretation of the data could have been further enhanced by the inclusion of fatty acids in maternal plasma phospholipids. Previous studies have examined the relationship between the maternal dietary intake of fatty acids and their plasma concentrations, demonstrating that both dietary intake and supplementation during pregnancy can modulate maternal plasma fatty acid levels. Furthermore, strong correlations have been documented between the LC-PUFA content of umbilical cord plasma phospholipids and that of maternal plasma phospholipids [44,45]. These findings support the notion that the maternal fatty acid status plays a key role in determining placental transfer and fetal plasma fatty acid composition [46]—particularly for DHA and, to a lesser extent, ARA. However, maternal ARA intake during pregnancy does not appear to significantly influence fetal plasma ARA levels, suggesting a degree of fetal-autonomous regulation. This interpretation is consistent with the findings of the present study, which identified no association between maternal ARA intake and placental phospholipid ARA levels but did reveal a positive association between maternal DHA intake and DHA concentrations in placental phospholipids. Another limitation of this explorative study was that we could not determine the number of subjects studied based on a concrete initial hypothesis. However, the result of a strong association between intake and placenta percentage content only for DHA but not for other FAs is not considered to be a chance finding due to limited sample size. In fact, a post hoc power analysis was conducted using R package “pwr” (version 3.3.3) to evaluate whether the sample size (*n* = 54) was adequate to detect meaningful effects. For a moderate correlation (r = 0.37), the analysis indicated a power of 80% (α = 0.05). This suggests that this study was sufficiently powered to detect medium-sized effects.

## 5. Conclusions

The placental transfer of both ARA and DHA is essential for fetal development [47]. In the present study, we observed a strong and statistically significant association between maternal dietary DHA intake and its concentration in placental phospholipids; however, no such association was found for other fatty acids, including ARA. Although total energy and fat intake during pregnancy are known to have important metabolic and health implications, they did not appear to influence placental DHA status. Similarly, maternal diet did not substantially affect placental phospholipid levels of the other fatty acids assessed. Nevertheless, the intake of these fatty acids may still be relevant for other health outcomes, such as offspring cardiovascular risk. Given the observational nature of this study and the relatively small sample size, these findings should be interpreted with caution. Nonetheless, they contribute valuable insight into the relationship between maternal diet and placental fatty acid composition and underscore the need for future research examining how maternal dietary patterns influence placental fatty acid profiles in relation to both maternal and neonatal plasma levels.

## Figures and Tables

**Table 1:** General characteristics of the PREOBE “mother–infant pairs” included in the present study, *n* = 54.

Characteristic	Mean ± SD *	Median (Percentile 25th, 75th) **	*n* (%) ***
Maternal age (years) *	32.0 ± 4.0	33 (29, 35)	
Pregestational BMI (kg/m^2^) **	24.5 ± 4.0	23.8 (21.6, 26.5)	
GWG (kg) *	10.9 ± 5.8	11.0 (8.0–14.0)	
GDM			15.12 (28)
Placenta weight (g) *	507 ± 143	500 (400, 600)	
Birth weight (g) *	3331 ± 461	3320 (3040, 3610)	
Infant’s sex			
Male			25 (46.3%)
Female			29 (53.7%)
Physical activity +			
Low active			20 (39.2%)
Active			25 (49.0%)
Very active			6 (11.8%)
Smoking before pregnancy +			
Yes			14 (27.5%)
No			37 (72.5%)

SD—standard deviation; BMI—body mass index; GWG—gestational weight gain; GDM—gestational diabetes mellitus. + Three cases did not report this variable *n* = 51. * Normally distributed continues variables (mean ± standard deviation). ** Non-normally distributed continuous variables (median and 25th, 75th percentiles). *** Categorical or nominal variables, number and percentage of the total sample.

**Table 2:** Maternal dietary intake of energy, macronutrients, and fatty acids at 34 weeks gestation, *n* = 54.

Nutrient	g/Day	% Energy	% Fat
Energy (kcal/d)	2019 ± 527 (1020–4080)		
Carbohydrates	221 ± 59 (96.5–404.0)	44.3 ± 7.6 (29.93–65.87)	
Protein	85 ± 22 (44.7–162.0)	17.0 ± 2.6 (23.03–25.23)	
Fat	87 ± 35 (30.6–236.0)	38.6 ± 7.7 (19.89–56.70)	
Saturated fatty acids	32.8 ± 17.5 (14.8–103.0)	14.0 ± 3.9 (7.45–24.73)	35.9 ± 5.9 (23.91–48.37)
Myristic acid (C14:0)	3.1 ± 2.2 (0.95–13.1)	1.3 ± 0.62 (0.54–3.07)	3.3 ± 1.3 (1.53–6.86)
Palmitic acid (C16:0)	16.2 ± 7.9 (6.4–48.1)	7.0 ± 1.87 (3.34–12.21)	18.2 ± 3.0 (10.34–23.20)
Stearic acid (C18:0)	6.5 ± 3.2 (2.3–18.2)	2.86 ± 0.85 (1.36–5.42)	7.4 ± 1.5 (3.88–11.64)
Monounsaturated fatty acids	36.2 ± 14.3 (9.2–84.1)	15.8 ± 4.2 (5.98–28.28)	41.08 ± 5.9 (30.07–54.60)
Palmitoleic acid (C16:1)	1.4 ± 0.63 (0.68–4.2)	0.65 ± 0.16 (0.32–1.01)	1.7 ± 0.35 (1.09–2.63)
Oleic acid (C18:1*n*-9)	32.5 ± 12.7 (7.5–74.2)	14.4 ± 4.17 (4.88–25.52)	37.1 ± 6.1 (24.51–52.34)
Polyunsaturated fatty acids	12.5 ± 5.2 (3.3–32.7)	5.4 ± 1.3 (2.15–9.97)	14.2 ± 3.0 (8.33–22.71)
Linoleic acid (C18:2*n*-6)	10.1 ± 4.3 (2.8–28.3)	4.4 ± 1.2 (1.82–8.85)	11.7 ± 2.8 (6.09–18.70)
α-Linolenic acid (C18:3*n*-3)	1.20 ± 0.48 (0.48–2.5)	0.55 ± 0.15 (0.29–0.98)	1.4 ± 0.4 (0.82–3.06)
Arachidonic acid (C20:4*n*-6)	0.14 ± 0.10 (0.008–0.71)	0.06 ± 0.03 (0.01–0.18)	0.17 ± 0.09 (0.03–0.45)
Eicosapentaenoic acid (C20:5*n*-3)	0.12 ± 0.11 (0.0–0.53)	0.06 ± 0.05 (0.0–0.19)	0.15 ± 0.13 (0.00–0.47))
Docosahexaenoic acid (C22:6*n*-3)	0.26 ± 0.20 (0.0–0.88)	0.12 ± 0.09 (0.0–0.40)	0.32 ± 0.25 (0.00–1.22)

All data are expressed as mean ± SD (min, max).

**Table 3:** Descriptive data of the composition of placental phospholipid fatty acid, *n* = 54.

Fatty Acid	Mean	SD
C14:0	0.37	0.09
C16:0	26.31	1.04
C17:0	0.32	0.05
C18:0	11.88	0.92
C20:0	0.30	0.09
C22:0	1.27	0.29
C24:0	1.43	0.44
C14:1	0.01	0.01
C15:1	0.05	0.01
C16:1*n*-7	0.42	0.10
C17:1	0.08	0.22
C18:1*n*-9	9.18	1.13
C18:1*n*-7	1.65	0.18
C20:1*n*-9	0.19	0.06
C22:1*n*-9	0.21	0.14
C24:1*n*-9	1.45	0.46
C14:1t	0.02	0.02
C16:1T	0.24	0.11
C18:1t	0.07	0.05
C22:1t	0.03	0.04
C18:2tt	0.05	0.02
C20:3*n*-9	0.13	0.05
C18:2*n*-6	9.32	1.04
C18:3*n*-6	0.12	0.04
C20:2*n*-6	0.43	0.09
C20:3*n*-6	4.59	0.87
C20:4*n*-6	21.64	1.54
C22:2*n*-6	0.24	0.05
C22:4*n*-6	1.56	0.26
C22:5*n*-6	1.07	0.44
C18:3*n*-3	0.02	0.01
C18:4*n*-3	0.05	0.06
C20:3*n*-3	0.11	0.03
C20:5*n*-3	0.08	0.07
C22:5*n*-3	0.46	0.33
C22:6*n*-3	4.64	0.76

All data are expressed as weight-% of total fatty acids measured.

**Table 4:** Correlations between maternal dietary fatty acid intake at 34 weeks of gestation and fatty acids in percentages of total placental phospholipids, *n* = 54.

Fatty Acids in Placental Phospholipids	FA Intake (g/Day) r Values	Energy (%) r Values	Fat (%) r Values
Total saturated fatty acid	0.097	0.154	0.050
Myristic acid (C14:0)	0.276 *	0.259	0.188
Palmitic acid (C16:0)	−0.091	0.033	0.059
Stearic acid (C18:0)	0.278 *	0.227	0.161
Palmitoleic acid (C16:1)	0.015	−0.093	−0.107
Oleic acid (C18:1*n*-9)	0.243	0.331 *	0.315 *
Linoleic acid (C18:2*n*-6)	0.139	0.110	0.216
α-Linolenic acid (C18:3*n*-3)	0.100	0.066	0.286 *
Arachidonic acid (C20:4*n*-6)	0.142	0.208	0.197
Eicosapentaenoic acid (C20:5*n*-3)	0.002	−0.043	−0.057
Docosahexaenoic acid (C22:6*n*-3)	0.469 ***	0.435 **	0.429 **

FA in placental phospholipids are expressed as percentage of total fatty acids. r—Pearson’s correlation coefficient. * Significant correlation *p* < 0.05. ** Significant correlation *p* < 0.01. *** Significant correlation *p* < 0.001.

## Data Availability

The original contributions presented in this study are included in the article. Further inquiries can be directed to the corresponding authors.

## Abbreviations

The following abbreviations are used in this manuscript:

ALA	α-linolenic acid
ARA	Arachidonic acid
BMI	Body mass index
DHA	Docosahexaenoic acid
EPA	Eicosapentaenoic acid
FA	Fatty acid
FFQ	Food frequency questionnaire
GDM	Gestational diabetes mellitus
GWG	Gestational weight gain
LA	Linoleic acid
LC-PUFA	Long-chain polyunsaturated fatty acids
MUFA	Monounsaturated fatty acid
PL	Phospholipids
SFA	Saturated fatty acid
